# Supplementary material for: Physiologic and Transcriptomic Effects Triggered by Overexpression of Wild Type and Mutant DNA Topoisomerase I in Streptococcus pneumoniae
Source: Int J Mol Sci. 2023 Oct 31;24(21):15800. doi: 10.3390/ijms242115800 (PMC10648598; doi:10.3390/ijms242115800)
Supplement: Supplementary file 1 [file ijms-24-15800-s001.zip › ijms-2648892-supplementary.pptx]

## Slide 1
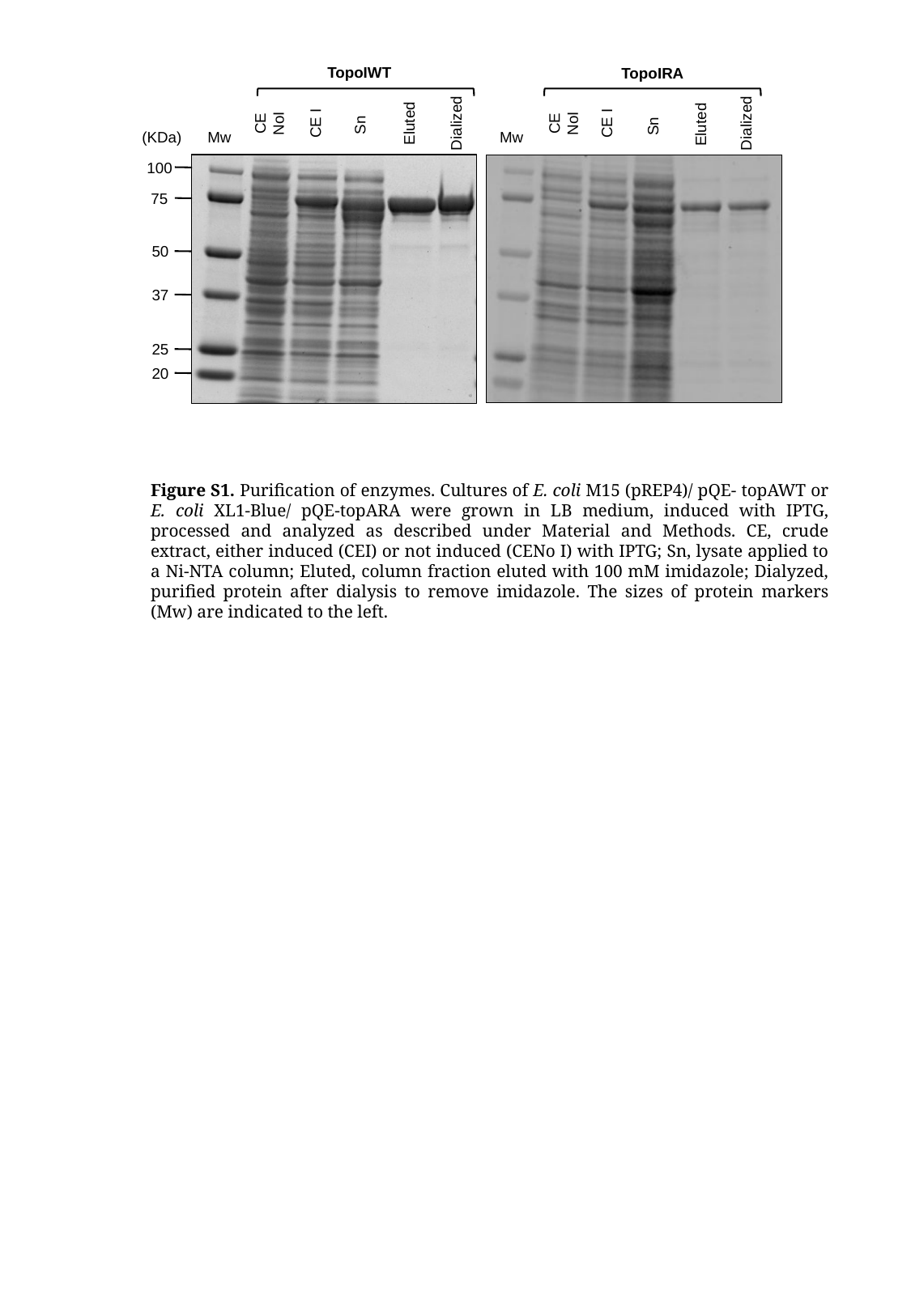

Sn
Eluted
Dialized
TopoIWT
CE I
CE NoI
Dialized
Sn
Eluted
TopoIRA
CE I
CE NoI
(KDa)
Mw
Mw
100
75
50
37
25
20
Figure S1. Purification of enzymes. Cultures of E. coli M15 (pREP4)/ pQE- topAWT or E. coli XL1-Blue/ pQE-topARA were grown in LB medium, induced with IPTG, processed and analyzed as described under Material and Methods. CE, crude extract, either induced (CEI) or not induced (CENo I) with IPTG; Sn, lysate applied to a Ni-NTA column; Eluted, column fraction eluted with 100 mM imidazole; Dialyzed, purified protein after dialysis to remove imidazole. The sizes of protein markers (Mw) are indicated to the left.

## Slide 2
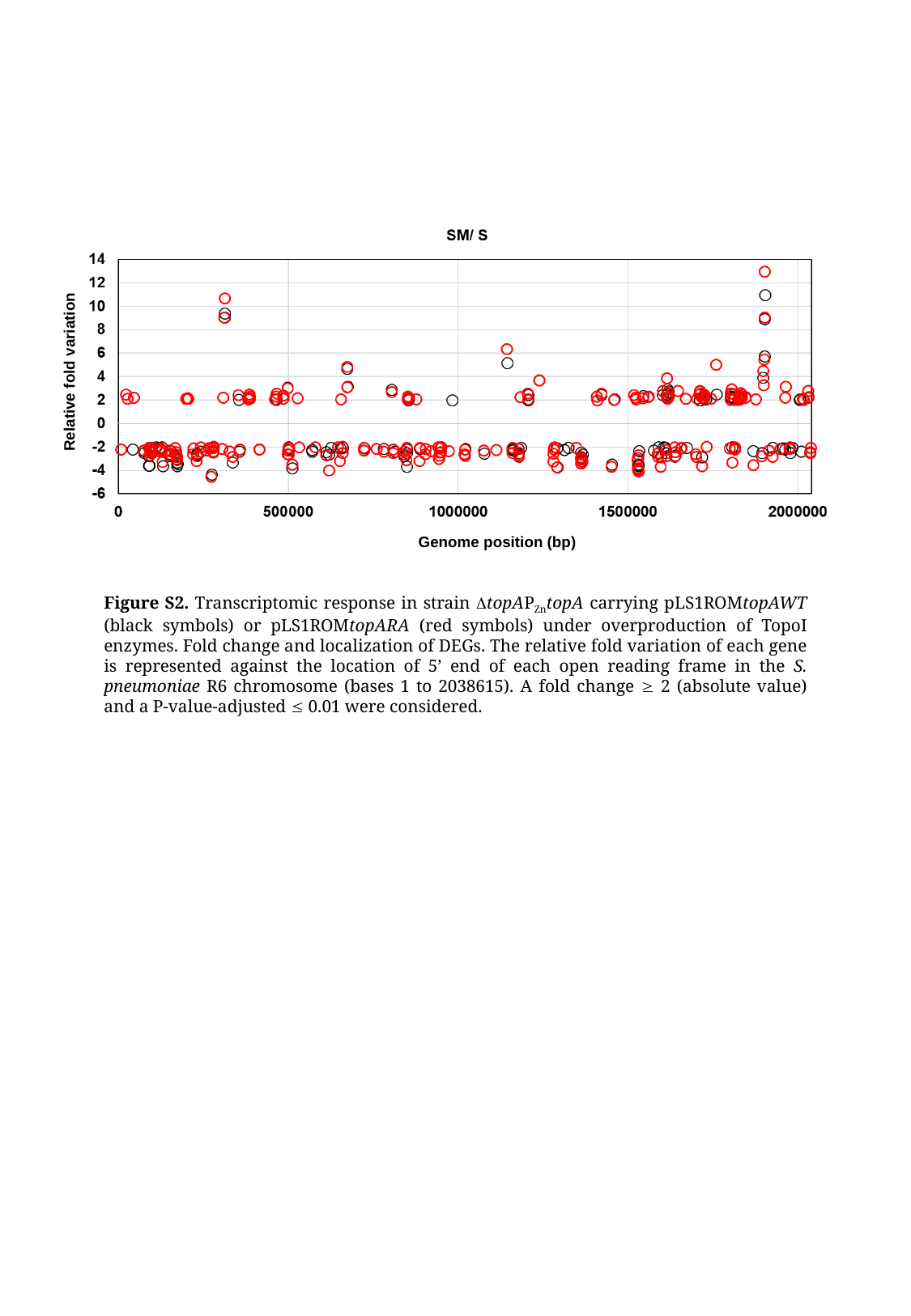

Relative fold variation
Genome position (bp)
Figure S2. Transcriptomic response in strain ΔtopAPZntopA carrying pLS1ROMtopAWT (black symbols) or pLS1ROMtopARA (red symbols) under overproduction of TopoI enzymes. Fold change and localization of DEGs. The relative fold variation of each gene is represented against the location of 5’ end of each open reading frame in the S. pneumoniae R6 chromosome (bases 1 to 2038615). A fold change ≥ 2 (absolute value) and a P-value-adjusted ≤ 0.01 were considered.

## Slide 3
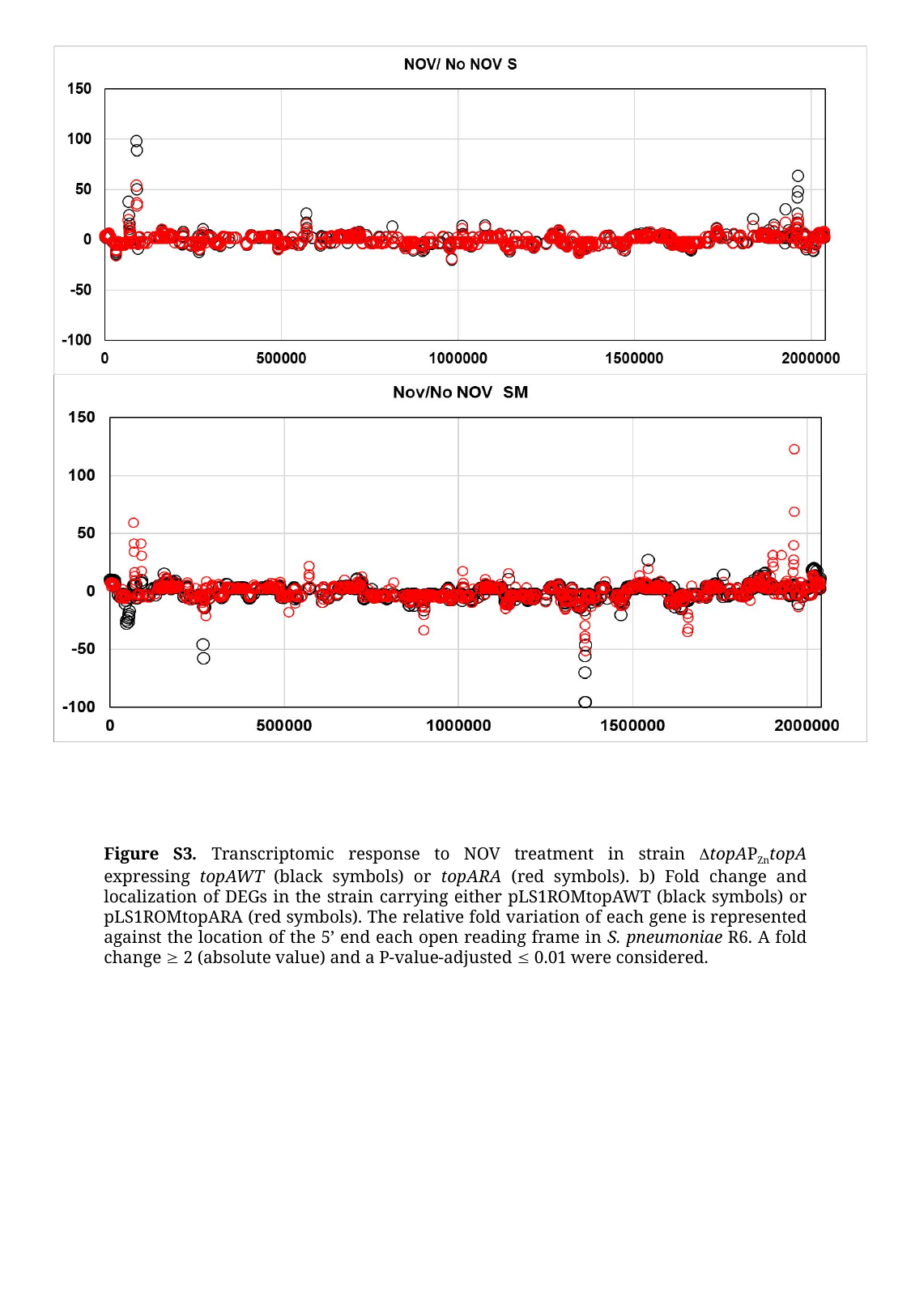

Figure S3. Transcriptomic response to NOV treatment in strain ΔtopAPZntopA expressing topAWT (black symbols) or topARA (red symbols). b) Fold change and localization of DEGs in the strain carrying either pLS1ROMtopAWT (black symbols) or pLS1ROMtopARA (red symbols). The relative fold variation of each gene is represented against the location of the 5’ end each open reading frame in S. pneumoniae R6. A fold change ≥ 2 (absolute value) and a P-value-adjusted ≤ 0.01 were considered.
